# The Rise of Remimazolam: A Review of Pharmacology, Clinical Efficacy, and Safety Profiles

**DOI:** 10.7759/cureus.57260

**Published:** 2024-03-30

**Authors:** Saiesh Dessai, Sanjot Ninave, Amol Bele

**Affiliations:** 1 Anesthesia, Datta Meghe Institute of Higher Education & Research, Wardha, IND

**Keywords:** balanced anesthesia, general anaesthesia, flumazenil, procedural sedation and analgesia, sedation, remimazolam

## Abstract

Anesthesiologists often use benzodiazepines (BZDs) due to their remarkable amnestic and anxiolytic capabilities. Because of this, they are perfect for use during the perioperative phase, when patients' anxiety levels are already high. Remimazolam has replaced certain commonly used intravenous (IV) anesthetics due to its excellent safety profile, rapid onset of action, and short half-life. The four classes of BZDs, 2-keto-benzodiazepines, 3-hydroxy-benzodiazepines, triazolobenzodiazepines, and 7-nitro-benzodiazepines based on chemical structure, provide various levels of drowsiness, forgetfulness, and anxiolysis. Based on their elimination half-life, short-acting BZDs typically have a half-life ranging from one to 12 hours, e.g., oxazepam; intermediate-acting BZDs have an average elimination half-life of 12 to 40 hours, e.g., alprazolam; and long-acting BZDs have an average elimination half-life of more than 40 hours, e.g., diazepam. The chloride ion channel is conformationally shifted by the benzodiazepine molecule resulting in central nervous system (CNS) inhibition and hyperpolarization. Each type of benzodiazepine has a favored use. For example, diazepam is used to treat anxiety. Midazolam is used for its anxiolytic and anterograde amnestic effects during the perioperative phase. Anxiety and epilepsy are two conditions that lorazepam effectively treats. There are now phase II and III clinical studies investigating remimazolam. It is not sensitive to alterations in its surroundings and has a brief half-life so that it may be removed rapidly, even after extensive infusion. Being a soft drug means the body easily breaks it down via metabolism, which explains many features. Remimazolam is hydrolyzed into methanol and its carboxylic acid metabolite CNS 7054 by esterase metabolism. Therefore, remimazolam has a shorter onset time and faster recovery than other BZDs. Remimazolam is metabolized independently of any particular organ. Patients with hepatic and renal problems will not see any changes in metabolism or excretion since the drug's ester moiety makes it a substrate for general tissue esterase enzymes. Like its predecessor, midazolam, it has a high potential for addiction. Some side effects that could occur during infusion include headaches and drowsiness. In clinical trials, hypotension, respiratory depression, and bradycardia were noted in participants. BZDs are helpful when used in conjunction with anesthesia. Remimazolam stands out, thanks to its unique pharmacokinetics, pharmacodynamics, safety profile, and potential medical applications. Its desirable properties make it a potential surgical premedication and sedative in the critical care unit. Anesthesiologists and other doctors could have access to more consistent and safer medication. However, additional comprehensive clinical trials are necessary to understand remimazolam's advantages and disadvantages.

## Introduction and background

An ultra short-acting benzodiazepine (BZD), remimazolam besylate is known by many names: Byfavo, Anerem, and Aptimyda^TM^ in Europe; Yufavo^TM^ in the United States; and Ruima in China. It was just given the go-ahead to be used as an anesthetic in Japan in January 2020. Furthermore, it has been authorized for procedural sedation in the United States in 2020, in China in 2020, in Europe in 2021, and in South Korea in 2021, all of which target adult patients [1]. The BZD family of medicines modulates chloride channels by blocking polysynaptic pathways and directly interacts with gamma-aminobutyric acid (GABA) [2]. BZDs are excellent sedatives in the context of anesthesia due to their anxiolytic properties; they reach a state that is something between being naturally relaxed and being asleep from general anesthesia [3]. Remimazolam increases the activation of GABA receptors, causing the cell membrane to become hyperpolarized and impairing neural function by encouraging an increased input of chloride ions [4]. Remimazolam provides several benefits over short-acting sedative medications already on the market [5]. These include an immediate onset of action, an independent metabolism that does not depend on any particular organs, a reversal agent, a brief duration of action, a predictable recovery, and a safety profile with hemodynamic stability comparable to other BZDs.

Aim

We aim to review the study and evaluate the pharmacological mechanisms, clinical efficacy, and safety profile of remimazolam; the comparative analysis with other existing sedatives or anesthetics; the emerging trends and future directions; and patient outcomes, addressing challenges and limitations.

Search methods for the identification of studies

Search Strategy

A comprehensive literature search was carried out through PubMed and CENTRAL using the keywords remimazolam and sedation, which were all part of our search technique, with topic headers and free-text phrases separated by the Boolean operators AND and OR between January 2017 and January 2023. We additionally searched for key references from bibliographies of the relevant studies published in English. Based first on the title and abstract and then on the complete texts, one of the reviewers independently tracked the retrieved studies against the inclusion criteria. About 20% of these papers were evaluated by another reviewer to confirm their inclusion. Discussions were used to settle disagreements. Exclusion criteria frequently include things like significant language impairments, psychological disorders like anxiety, and other physical issues like obesity, cardiovascular ailments, etc.

Selection Criteria

**Figure 1:**
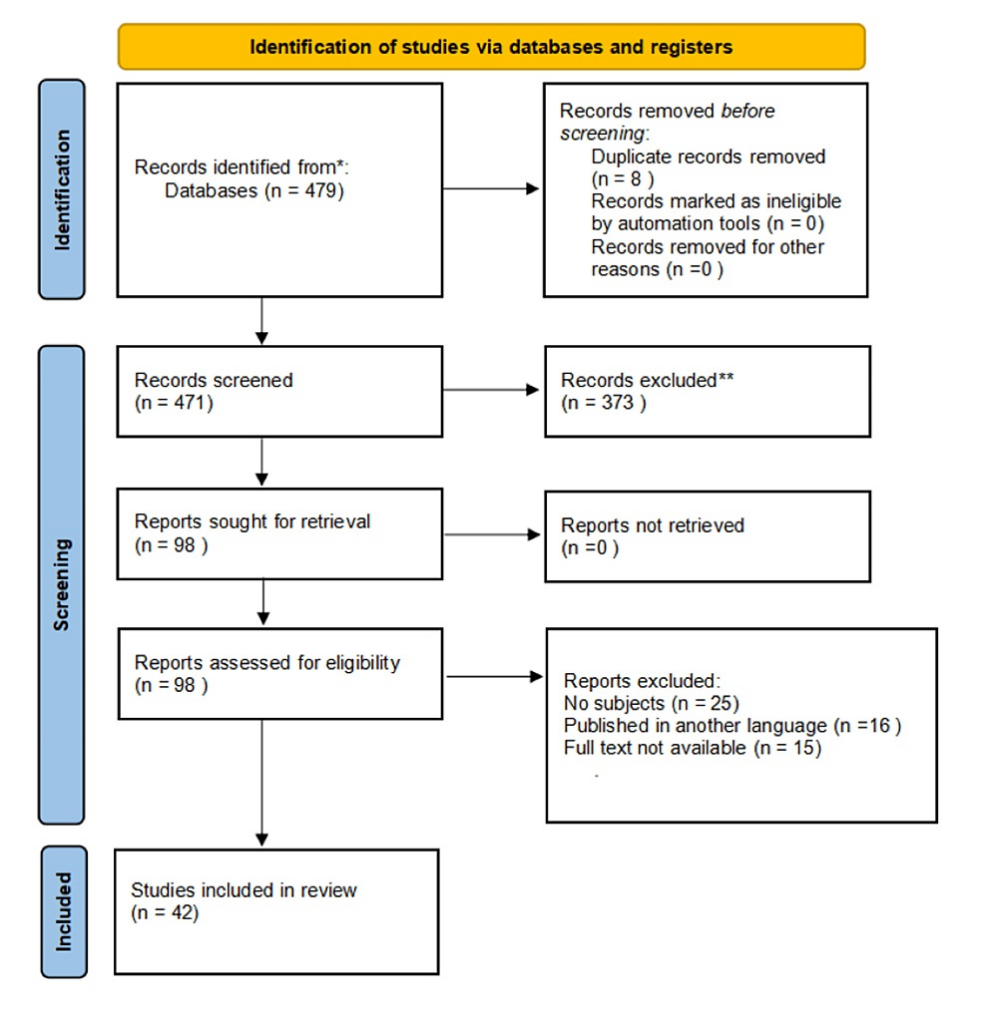
Research methodology using the PRISMA method PRISMA: Preferred Reporting Items for Systemic Reviews and Meta-Analysis

Retrospective and prospective cohort studies, randomized and nonrandomized human-controlled trials, and systematic reviews were among the selection criteria. Technical reports, editorials, animal studies, cadaver studies, and review pieces were eliminated due to resource constraints or situations where reviewers could not access the entire text; we did not include the research published in other languages (Figure 1).

Search Outcomes

A total of 479 articles that matched the requirements were discovered during the initial search. A total of 373 studies were excluded because they were irrelevant, while another eight were excluded because they were duplicates. After reading the complete text of the publications, 56 were excluded because of missing crucial data. In the end, 42 papers satisfied the criteria to be included in the study.

## Review

Characteristics

Remimazolam is a gamma-aminobutyric acid (GABA-A) receptor short-acting agonist with the chemical formula C21H19BrN4O2 and an average mass of 439.305 Dalton (Da). It has an ester moiety and a structure similar to midazolam. Because it dissolves in water, injection sites experience less pain [6]. It quickly becomes an inactive molecule in its initial state due to metabolic activities [7]. Midazolam is the parent molecule from which remimazolam is made. Because it has a carboxylic ester bond, nonspecific tissue esterases may break down the drug. Remimazolam, the newest "soft medication," is based on the molecular formula of midazolam. It features an extra ester side chain.

Mechanism of action

Specifically, remimazolam interacts with GABA-A receptors [8]. The primary inhibitory neurotransmitter in the brain and spinal cord is GABA. Anxiolytic effects at low doses and sedative/hypnotic effects at high dosages are the result of the dosage-dependent action of BZDs on GABA receptors [9]. There is no specific organ that is required for its rapid metabolism and high clearance rate. Carboxylesterase 1A (CES 1A), mostly found in human livers, is a crucial nonspecific esterase that plays a role in this process and transforms it into CNS 7054. The binding affinity of this metabolite, known as an inactive form, at the GABA-A type A receptors is around 300 to 400 times lower [10]. Because it is the primary inhibitory receptor, the GABA receptor is primarily targeted by intravenous (IV) anesthetics that bring about general anesthesia. Quick biotransformation into inactive metabolites is the goal of a specific structural design among IV anesthetic medicines.

Metabolism

Remimazolam is hydrolyzed into methanol and its carboxylic acid metabolite, CNS 7054, by esterase metabolism. When exposed to liver homogenates from humans, rats, mice, and mini-pigs, the acid metabolite CNS 7054 is rapidly produced from remimazolam (base, CNS 7056X), according to initial findings [11]. It is often stated that carboxylesterase 1 (CES 1) is the principal esterase that metabolizes remimazolam [6,12]. The esterase converts remimazolam to CNS 7054, its principal inactive metabolite, which has a 400-fold decrease in affinity for the GABA-A receptor [13]. Several medications rely on CES 1 for their metabolism, including methylphenidate, clopidogrel, and activating angiotensin-converting enzyme inhibitor prodrugs [14].

Adverse effects

Hypotension, vomiting, and nausea are the most prevalent adverse responses to anesthesia [15-17]. Remimazolam groups showed less injection site discomfort, fewer cases of hypotension, and less need to manage bradycardia compared to propofol groups. There may be a small benefit to propofol in postoperative nausea and vomiting (PONV) [16]. There is a risk of misuse, and tolerance and dependency may develop with long-term use of BZDs. Although its brief half-life and IV administration requirements help to alleviate these worries, remimazolam is nevertheless likely to shoulder some of these responsibilities.

Procedural sedation

In a phase Ib dose-ranging experiment with healthy volunteers, remimazolam besylate was first used for colonoscopy for the first time [18]. Sedation is administered to patients to alleviate fear, pain, and memory loss, making complex diagnostic procedures, such as endoscopy, more tolerable [19]. Tissue and plasma esterases are readily available and metabolize remimazolam quickly. Its elimination clearance and body weight have little effect on its first-order pharmacokinetic profile, and its metabolism is independent of organs. However, despite these characteristics, it has a safety profile that is on par with midazolam [20]. Fear, anxiety, and discomfort, whether mental or physical, are everyday experiences for patients before, during, or after clinical procedures [21]. Systemic issues might emerge as a result of this anguish.

The American Society of Anesthesiologists defines a moderate level of sedation as a level at which a sedated patient may respond voluntarily to verbal or tactile stimuli [22]. Suppose the patient "responds only when (his/her) name is screamed loudly or repeatedly [23]," as measured by the Modified Observer's Assessment of Alertness/Sedation scale. In that case, they are appropriately sedated, according to prior studies. The sleepiness that remimazolam causes is dose-dependent and takes action within 60 seconds after the dose [24]. Results from a phase IIa clinical trial comparing remimazolam with midazolam for procedural sedation during esophageal and gastric endoscopy showed that the former had a quicker recovery time.

General anesthesia

Despite their efficacy as induction medications, BZDs have not seen extensive usage due to issues with control over anesthesia level [8], slow hypnotic effects [25,26], and delayed consciousness recovery. It will take at least one to three minutes after ingestion before the effects of 0.075 mg/kg become apparent [27]. The efficacy of remimazolam under general anesthesia was the subject of a large body of clinical research. If you are looking for a safer alternative to propofol for generating and maintaining general anesthesia, try total intravenous anesthesia (TIVA) using IV remimazolam as the hypnotic component [16]. Remimazolam was administered intravenously at 6 or 12 mg/kg/h doses during induction (n = 150 for each dosage). Separately, 75 patients were given a standard IV propofol dosing regimen, which included 2.0-2.5 mg/kg/h initially and 4-10 mg/kg/h after that. All patients were administered remifentanil at a rate ranging from 0.25 to 0.5 μg/kg/min for the experiment. No rescue sedative was necessary, no intraoperative awakening or memory loss, and no bodily movement were the three key efficacy goals that all three patient groups met, thereby achieving noninferiority. On average, patients needed significantly more time to reach unconsciousness when administered with remimazolam at doses of 6 mg/kg/h and 12 mg/kg/h (102.0 s and 88.7 s, respectively) compared to propofol (78.7 s). Similarly, the extubation times for remimazolam (both doses) were notably longer at 19.2 minutes compared to 13.1 minutes for propofol [16]. Remimazolam regimens had a far better safety profile. The remimazolam group had a lower incidence of hypotensive episodes. The requirement of vasopressor support was less compared to propofol. Both induction regimens were equally safe and efficacious in high-risk surgical patients [28]. Overall, the early clinical results of remimazolam's use as a component of general anesthesia show a very encouraging efficacy. When selecting for general anesthesia, it is essential to consider remimazolam's more excellent hemodynamic stability (Table 1).

**Table 1:** Analysis of the studies

Author	Year	Dose of remimazolam	Result
Luo et al. [29]	2022	"Comparative Study About Different Doses of Remimazolam in Short Laparoscopic Surgery"	Even after controlling for propofol, the median group still required more time for surgical extubation than the high group.
Matsumoto et al. [30]	2023	“Remimazolam's Effects on Postoperative Nausea and Vomiting Are Similar to Those of Propofol”	Remimazolam and propofol have comparable antiemetic effects, as there was no discernible difference under general anesthesia.
Katsuragawa et al. [31]	2023	“Effect of remimazolam versus sevoflurane on intraoperative hemodynamics in noncardiac surgery”	Cumulative hypotension time, incidence of vasopressor usage, and dosage of ephedrine were all decreased when anesthesia was maintained with remimazolam.
Cui et al. [32]	2023	“Efficacy and Safety of Remimazolam Tosilate Combined with Propofol in Digestive Endoscopy: A Randomised Trial”	Painless digestive endoscopy compared standard propofol with a new intravenous sedative called remimazolam tosylate. Combining remimazolam tosylate with propofol provides an anesthetic alternative for painless endoscopies that is virtually as safe as propofol alone, according to the research. This combination has a low rate of adverse effects.
Hu et al. [33]	2022	“Remimazolam: An Updated Review of a New Sedative and Anaesthetic”	The new intravenous benzodiazepine remimazolam has received approval for use in general anaesthesia and procedural sedation. High procedure success rates, rapid onset and recovery, and reversibility with flumazenil are some of its notable features.
Sneyd et al. [34]	2021	“Current status of perioperative hypnotics, role of benzodiazepines, and the case for remimazolam: a narrative review”	Hypotension, high-dose propofol, and benzodiazepines are driving up the need for sedation, which is putting an additional strain on anesthesiologists and sedationists. Anesthesia may be achieved by combining remimazolam with remifentanil.
Sneyd & Rigby-Jones [35]	2020	“Remimazolam for anaesthesia or sedation”	Compared to midazolam, remimazolam has better recovery times and success rates for procedural sedation. It may also be helpful as a hypnotic component in general anesthesia and for evaluating anticonvulsants.
Wang et al. [36]	2022	“Profile of Remimazolam in Anesthesiology: A Narrative Review of Clinical Research Progress”	Remimazolam is a short-acting GABA receptor agonist with great promise for various medical uses, such as sedation and general anesthesia. A potential contender for comfort diagnostics and fast-track surgery, it has inactive metabolites and weak interactions with other medications.
Yeh et al. [37]	2023	“Remimazolam as a Primary Agent for Brief Invasive and Noninvasive Procedures: A Case Series”	Seven patients, ranging in age from 14 to 51, were mostly sedated for procedures using Remimazolam, a new benzodiazepine with sedative and amnestic characteristics. Results showed that low-intensity treatments were sufficient to address variations in oxygen saturation and blood pressure.

The role of flumazenil

As a BZD receptor antagonist, flumazenil (Romazicon) is well-known in the medical community. A benzodiazepine-3-carboxylate, flumazenil is ethyl 8-fluoro-5,6-dihydro-5-methyl-6-oxo-4H-imidazo[1,5-a] in its chemical formula (1,4). As an imidazobenzodiazepine, flumazenil has a molecular formula of 303.3. It blocks the action of BZD and non-BZD medications by binding to the GABA/BZD receptor complex's BZD receptor site. Additionally, the affinity of BZDs for BZD receptors may be inverted. You will not feel the effects for around a minute or two [38]. Typical doses range from 0.1 to 0.2 mg, and peak plasma levels are 3 to 6 ng/mL. The reversal effects of the drug should begin to take action within a minute or two after injecting it. To achieve the desired degree of consciousness, inject 0.2 mg again 60 seconds after the initial dose; then, inject 1 mg every 60 seconds [39]. Once plasma flumazenil concentrations decrease, remimazolam might be used for repetitious sedation. It is essential to properly monitor patients after administering flumazenil for any indications of re-sedation [40].

## Conclusions

Remimazolam is well-tolerated due to its rapid onset and rapid offset. Remimazolam presents advantages over propofol due to its favorable safety profile, characterized by decreased incidence of cardiovascular and respiratory depression and the presence of a reversal agent. The qualities above provide an advantageous IV anesthetic for various operations. It may take some time to replace our more well-known IV anesthetics, which are now just in the clinical trial stage. Still, it might provide us with more predictability and control over our anesthetic. Some potential adverse effects include uncontrollable movement during infusions, somnolence after the procedure, and headaches. The patient's involuntary movement becomes a noticeable problem during several surgical procedures. Additional large-scale clinical studies are still necessary to help the medical community better comprehend its characteristics. New formulations would expand the applicability of remimazolam, particularly in scenarios where IV administration poses challenges.
